# Evaluating the effects of standardized hydroalcoholic extract of oat grains (*Avena sativa* L.) capsules as an adjunctive treatment on allergic rhinitis 

**DOI:** 10.22038/ajp.2025.26055

**Published:** 2026

**Authors:** Farzad Nasrpour Tahouneh, Mehdi Ansari Dogaheh, Fariba Sharififar, Fatemeh Dabaghzadeh, Nasrin Bazargan, Sarehossadat Ebrahimi, Tania Dehesh, Faraz Ahmad

**Affiliations:** 1 *Herbal and Traditional Medicines Research Center, Kerman University of Medical Sciences, Kerman, Iran.*; 2 *Pharmaceutics Research Center, Institute of Neuropharmacology, Kerman University of Medical Sciences, Kerman, Iran.*; 3 *Department of Clinical Pharmacy, Faculty of Pharmacy, Kerman University of Medical Sciences, Kerman, Iran.*; 4 *Department of Pediatrics, Faculty of Medicine, Kerman University of Medical Sciences, Kerman, Iran.*; 5 *Modeling in Health Research Center, Institute for Futures Studies in Health, Kerman University of Medical Sciences, Kerman, Iran.*; 6 *Department of Biotechnology, School of Bio Sciences and Technology (SBST), Vellore *; 7 *Institute of Technology, Vellore - 632014, India*

**Keywords:** Allergic rhinitis, Avena, Beta-glucans, Capsules

## Abstract

**Objective:**

Allergic rhinitis is a chronic inflammatory disease which exists throughout people's lives. *Avena sativa* L. (oat) belongs to the Poaceae family and has antihistamine, anti-inflammatory, and antioxidant effects. The aim of this study was to evaluate the effects of standardized hydroalcoholic extract of oat grains capsules as an adjunctive treatment on allergic rhinitis.

**Materials and methods:**

Hard gelatin capsules of the dried extract of oat grains were prepared, and characterized for key physico-chemical properties. Patients diagnosed with allergic rhinitis were recruited for the clinical trial to assess the effectiveness of these capsules, and were randomly divided into treatment and control groups. The participating subjects received one oat extract or placebo capsule twice daily for two weeks. All the subjects (in the control and treatment groups) also received oral antihistamines and glucocorticoid nasal spray as the standard treatment. The patients’ total nasal symptom scores and the presence of allergic rhinitis symptoms were recorded at the baseline and at the end of the second week.

**Results:**

After two weeks of intervention, the patients’ total nasal symptom scores and the prevalence of allergic rhinitis symptoms significantly decreased in all of them. However, supplementation with oat grain extract capsules was associated with significantly higher improvements in the outcomes in the treatment group compared with the control group.

**Conclusion:**

The dried extract of oat grains (*Avena sativa* L.) capsules was an effective adjunctive therapy to improve allergic rhinitis symptoms.

## Introduction

Allergic rhinitis is one of the most common global health concerns (Bachert et al. 2017), with a prevalence of around 40% which is on the rise (May and Dolen 2017). It is a chronic inflammatory disease associated with other inflammatory diseases such as asthma, rhinosinusitis, allergic conjunctivitis, otitis media, and eczema (Gough et al. 2015). Allergic rhinitis is usually caused by IgE-dependent allergic inflammation, and multiple cell types and inflammatory factors such as mast cells, CD4+T cells, B cells, macrophages, eosinophils, interleukins and histamine are involved in its pathogeneses (Small et al. 2018). Its common symptoms include sneezing, nasal itching, upper respiratory tract congestion, runny nose, eye itching and redness, tearing, conjunctivitis, post nasal drip, nasal polyps, cough, fatigue, and daily dysfunction (Bachert et al. 2017; May and Dolen 2017). Allergic rhinitis is classified as mild or moderate/severe according to symptoms severity. Symptoms are categorized as mild when the quality of life is not affected. Moderate/severe severity is defined as the presence of troublesome symptoms that cause sleep disturbance or impairment of daily activities, leisure and/or sport or impairment of work or school performance (Dykewicz et al. 2020). Notable to say that the economic impact of this illness is so significant that it should be taken into consideration (Bachert et al. 2017; May and Dolen 2017).

The purpose of allergic rhinitis treatment is to reduce or eliminate the symptoms as well as preventing the future attacks and complications (May and Dolen 2017). There are a variety of approaches to control this disease including patient education, avoidance of allergens, pharmacotherapy and immunotherapy (May and Dolen 2017). Corticosteroids, antihistamines, decongestants, antagonists of leukotriene receptors and anti-IgE monoclonal antibodies are some of the medications used in this respect. However, each type of drug has inevitable side effects, and there is a need to develop medications with fewer side effects and greater efficacy (Bachert et al. 2017; May and Dolen 2017). Furthermore, the patients with allergic rhinitis increasingly self-medicate with health supplements such as prebiotics, probiotics, vitamins, minerals, amino acids, and herbal and animal compounds because they are of low cost, available and perceived to be safe in spite of the fact that clinical studies undertaken on their efficacy and safety are limited (Pellow et al. 2020). Nevertheless, some clinical studies evaluated the effects of medicinal plants on allergic diseases (Gholamnezhad et al. 2019; Javid et al. 2019).

*Avena sativa* L. (Oat) belongs to the Poaceae family and grows in different parts of the world because of its adaptation to different climates. Oats are commonly consumed as whole grains which are rich sources of fibers, proteins, carbohydrates, minerals, vitamins, and other phytochemical substances such as alkaloids, flavonoids, phenolic compounds, saponins, tannins, quinones, carotenoids, and phytosterols (Sang and Chu 2017; Sood et al. 2022). In addition, the major compounds of oat grains are beta-glucan (soluble dietary fiber) and avenanthramides (phenolic alkaloids) that are responsible for many of oat beneficial effects such as decreasing the risk of type 2 diabetes, cancer, and cardiovascular diseases. These bioactive compounds also have potent anti-inflammatory, antioxidant and anti-proliferative properties (Kim et al. 2021; Sang and Chu 2017). Interestingly, avenanthramide has been shown to have therapeutic effects on asthma, atopic dermatitis, allergic conjunctivitis, pruritic skin diseases, and other allergic diseases (Perrelli et al. 2018). Likewise, beta-glucan has been reported to have potential anti-allergic properties (Jesenak et al. 2014a). It should also be noted that no side effects or toxicity has been observed in therapeutic doses of oats, and these available low-cost grains (Ibrahim et al. 2020) are safe in both children and adults, even in children with celiac disease (Al-Snafi 2015; Aparicio-García et al. 2021). 

People in some regions of Iran consume oat decoction for alleviation of allergic rhinitis symptoms. Hence, the aim of this study was to evaluate the beneficial effects of standardized hydroalcoholic extract of oat grains capsules as an adjunctive treatment on allergic rhinitis.

## Materials and Methods

### Preparation of plant samples and extraction

About 20 kg of grains (*Avena sativa* L.) were prepared from the market after approval in Pharmacognosy Department of Faculty of Pharmacy, Kerman University of Medical Sciences, Kerman, Iran. A voucher specimen of the plant grains was deposited in Herbariun Center (KF1208). After crushing and sieving with 35 mesh, the plant seeds were extracted with 50% ethanol by Soxhlet method and concentrated under vacuum conditions (El-burai et al. 2020). The yield of the extraction was 5 g/100 g dried plant materials.

### Total phenolic content determination

To standardize the plant, its total phenolic compounds were determined by the Folin-ciocalteau colorimetric method and based on the calibration curve of gallic acid (Y=0.0071X+0.151, R2= 0.9997) as a reference compound (Azizi et al. 2022). The quantity of the total phenolic compounds in the plant was found to be 7.21± 0.67 (w/w %). 

### Analytical method for validation of oat extract

A spectrophotometric method for the determination of oat extract λmax was developed and validated. The λmax of the oat extract in water was found to be 273 nm. Standard concentrations were prepared, and the absorbance of the solutions was measured at λmax (Azizi et al. 2022). The oat extract followed linearity in the concentration range 100–500 μg/ml by an equation of Abs= 0.0034*conc. - 0.0406 with the correlation coefficient of 0.9999 in water.

### Hard gelatin capsules formulation

To obtain the final formulation, the dried extract of oat grains was granulated by mixing it with corn starch and microcrystalline cellulose, and then, the granules were filled into hard gelatin capsules size 00 using the manually operated capsule filling machine (LMDTJ-C, Iran). The final formulation, comprising 300 mg of *A. sativa* L. extract, 150 mg of corn starch, and 150 mg of microcrystalline cellulose, should have a claimed weight of 600 mg.

### Characterizations of the prepared capsules formulation

#### Weight variation

Ten capsules of the final formulation were randomly picked and weighed using analytical scales. The results showed that all the capsules weights were in the acceptable range (90-110% of label claim) (Azizi et al. 2022). 

### Content uniformity

The oat extract content of 10 randomly selected capsules was determined by the absorbance measurement of the prepared samples at λmax and calculation of the percentage remained using the calibration curve equation. All the oat extract capsules content was in the acceptable range (85-115%) (Azizi et al. 2022).

### Dissolution rate

The dissolution profile of the prepared capsules formulation was determined using 500 ml of water with the temperature adjusted to 37℃ ± 0.5℃ as the dissolution medium, in the USP apparatus I (Basket, Erweka DT80, Germany) at the speed of 75 rpm, with the sampling times of 0, 5, 10, 15, 20, 30, 60, 90, 120, 150, 180, 240, 300, and 360 min (Mulia et al. 2019). The dissolved amount of the oat extract was measured spectrophotometrically, and the dissolved percentage was calculated for each capsule (n=3) at each time interval. The dissolution profile was constructed through the dissolved percentage versus time. 

### Stability study

The stability of the prepared capsules formulation packaged in tightly closed bottles with desiccants in the caps was determined through the measurement of the percentage of the oat extract remained by monitoring the oat extract content of the capsules stored at three different conditions including the refrigerator (2-8℃), environment (15-25°C), and oven (40℃ and relative humidity )75 ± 5%). The capsules specifications and the remaining percentage of the oat extract were measured at the intervals of 0, 1, 2, 8, 12, and 24 weeks (Azizi et al. 2022).

### Clinical evaluation

#### Study design, setting and ethical considerations 

This research was carried out as a randomized double-blind, placebo-controlled clinical trial (the physicians and the patients were blind) in the specialized clinic of Afzalipour Educational and Medical Center, Kerman, Iran from September 2021 to February 2022. This study was approved by the ethics committee of Kerman University of Medical Sciences under the number IR.KMU.REC.1398.354 and registered in Iranian Registry of Clinical Trials under the identity number IRCT20110310006026N12. Each participant read and signed an informed consent form before beginning the study.

### Inclusion and exclusion criteria

The individuals in the range of 12-65 years who had been diagnosed with allergic rhinitis by the physicians (based on clinical grounds) were included in the study. The exclusion criteria were pregnancy, nasal polyps, underlying medical diseases particularly autoimmune and celiac disease, and sensitivity to the 2nd generation antihistamines and oats. Medication non-adherence was defined as taking less than 80% of the prescribed medications (Manjit Singh et al. 2022). The patients with medications non-adherence were excluded from the study.

### Interventions

The participants were divided into control and treatment groups in a 1:1 ratio using block randomization with a block size of four. A person with no clinical involvement in the trial generated allocation sequence. For two weeks, the patients took one oat-extract capsule twice daily (each capsule containing about 300 mg of the extract equivalent to 21 mg of the phenolic compounds) or a placebo (like the oat extract capsule but containing an inert substance (starch) instead of an active ingredient) (Lee et al. 2004; Miraj and Kiani 2016). All the subjects (the control and treatment groups) also received the 2nd generation oral antihistamines (cetirizine) and glucocorticoid nasal spray as the standard treatment at the beginning of the study. 

### Outcome measurements

Based on Total Nasal Symptom Score (TNSS), each of the four main symptoms of allergic rhinitis including nasal itching, nasal congestion, runny nose and sneezing was given a score between 0 and 3 based on the severity of the symptoms (no symptoms 0, mild intensity symptoms 1, medium intensity symptoms 2, and high intensity symptoms 3) as reported by the patients. The total scores of these 4 symptoms were between 0 and 12 (Ellis et al. 2015; Lee et al. 2004). Moreover, the presence of allergic rhinitis symptoms including nasal congestion, rhinorrhea, sneezing, itchy nose, redness and itching of the eyes, tearing, headache, cough, anosmia, swelling and puffiness of the eyes, eye pain and burning, itchy face, blowing nose, dry throat in the morning, itchy throat and mouth, thirst, fatigue and halitosis (Dykewicz et al. 2020) were recorded. The clinical symptoms were evaluated at the baseline and at the end of the 2nd week. During this time, the patients were asked by phone about potential adverse effects and their compliance with the interventions. The patients who were unable to continue the treatment were excluded from the study. Also, the demographic information was recorded for all the patients including age, sex, education, place of living, weight, drug allergies, concomitant allergic diseases and family history of allergies. Furthermore, allergic rhinitis was classified into mild or moderate-severe according to the severity and into intermittent or persistent according to the duration of symptoms at the baseline (Brożek et al. 2010).

### Statistical analysis

The sample size was estimated based on the formula for comparing the two means. Considering the type 1 error (α) of 0.05 and type 2 error (β) of 0.2 (power = 80%), the sample size was estimated 17 patients in each group. The SD and the difference in the mean of the allergic rhinitis symptoms scores were estimated as 0.81 and 0.78, respectively (Lee et al. 2004; Nabavi et al. 2017). With 20% dropout rate, 21 participants were needed in each group. In order to confirm the 1st power (80%) pre hoc (a priori) power analysis (effect size of 0.05) was performed after data collection, and the same power (80%) was reached. This shows the acceptance of the results. The SPSS 22 software was used to analyze all of the data. The data distribution was normal based on Kolmogorov-Smirnov test. Independent samples t-test and chi-square test or Fisher's exact test were used to assess the difference between the quantitative and qualitative data, respectively. Mixed model analysis of variance was employed to investigate the differences in TNSS between the treatment and control groups over two weeks. Paired sample t-test was applied to compare the changes over time in TNSS in each group. The generalized estimating equation (longitudinal model) was used to compare the presence of allergic rhinitis symptoms between the treatment and control groups over the two weeks. For each symptom, the day 14 data was subtracted from the baseline data and then, new variables were defined. The obtained numbers in the new variable may be negative, zero, or positive, indicating improvement, no change, and deterioration of each symptom, respectively. A Chi-square test was performed to assess the new variable changes between the two groups. The percentage of change in each group was also calculated with the new variable. The p-value less than 0.05 was considered statistically significant. 

## Results

### Dissolution test 

**Figure 1:**
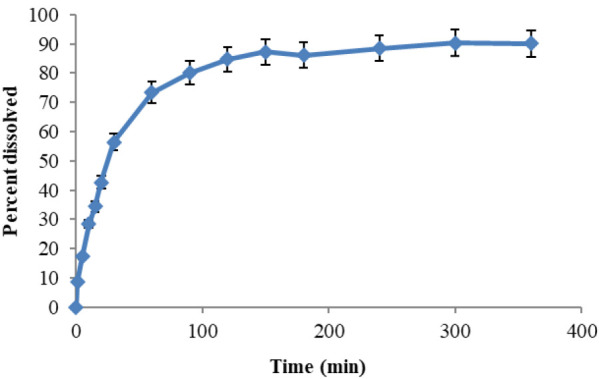
Dissolution profile of prepared oat extract capsules (Mean ± SD; n = 3), SD: standard deviation

The oat extract percentage dissolved over time is illustrated in Figure 1. The results depicted that more than 70% of the oat extract content of the capsules was dissolved in 60 min and more than 80% in 90 min. The results showed that the release rate gradually decreased with time. 

### Stability test

**Figure 2:**
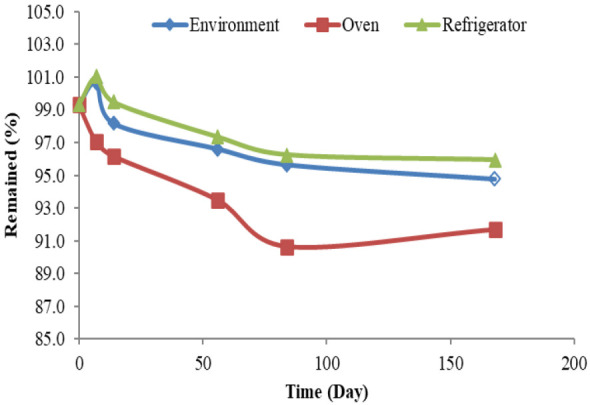
Stability of oat capsules formulation at different conditions up to 24 weeks

The remaining oat extract content (%) of the capsules in different time intervals and at three distinct temperatures (refrigerator, environment, and oven) is provided in Figure 2. The results showed that the content of the oat extract in the capsules stored in the oven was less than that of the capsules stored in other conditions; however, the remaining percentage of the oat extract of the capsules was more than 90% after 24 weeks in all the three conditions. Also, no changes in capsules specifications were observed in any of the three conditions during 24 weeks.

### Clinical findings

**Figure 3:**
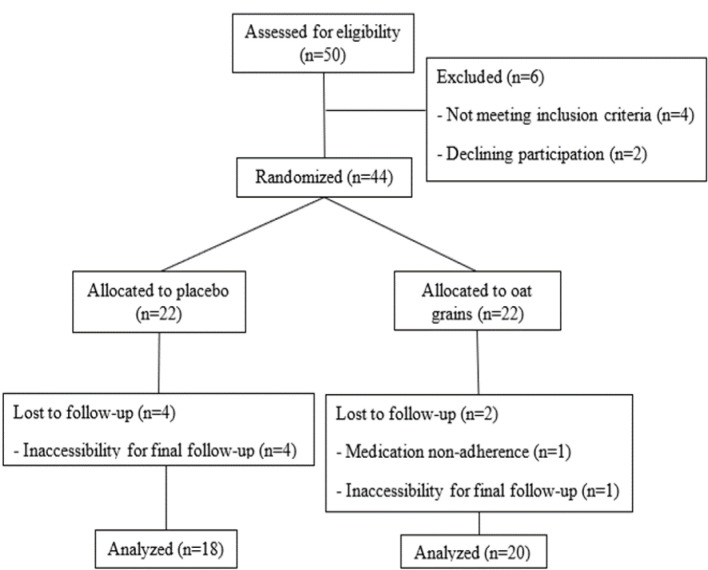
CONSORT flow diagram of participants in this clinical trial

Thirty-eight participants (20 in the treatment group and 18 in the control group) completed this trial. Figure 3 shows the flowchart of this study. The mean age of the patients was 19.29 ± 10.27 years old. There were 25 (65.79%) males and 13 (34.21%) females who participated in this study. Two patients (5.26%) (one in the treatment group and one in the control group) had allergic reactions to penicillin, a clinical factor that may be associated with allergic rhinitis. Among 38 participants, 11 ones were newly diagnosed with allergic rhinitis. The mean duration of disease was 48.96 ± 26.65 months in 27 patients previously diagnosed with allergic rhinitis. The demographic characteristics of the recruited participants are presented in Table 1. There was no significant difference related to the demographic information between the treatment and control groups.

**Table 1:** Demographic characteristics of participants in clinical trial in treatment and control groups

**p-value** ^*^	**Control (n=18)**	**Treatment (n=20) **	**Variables**
	**Number (Percent)**	**Number (Percent)**	
0.914	12 (66.67%) 6 (33.33%)	13 (65.00%) 7 (35.00%)	Sex Male Female
0.703	1 (5.56%) 13 (72.21%) 3 (16.67%) 1 (5.56%)	0 (0.00%) 14 (70.00%) 4 (20.00%) 2 (10.00%)	Education Illiterate High school diploma or less Bachelor's degree Master's degree or higher
0.602	8 (44.44%) 9 (50.00%) 1(5.56%)	9 (45.00%) 8 (40.00%) 3 (15.00%)	Place of living City Town Village
0.730	1 (5.56%) 17 (94.44%)	1 (5.00%) 19 (95.00%)	Drug allergies Yes No
0.106	10 (55.56%) 8 (44.44%)	16 (80.00%) 4 (20.00%)	Family history of allergies Positive Negative
0.17	4 (22.22%) 14 (77.78%)	1(5.00%) 19 (95.00%)	Concomitant allergic diseases Yes No
0.616	3 (16.66%) 15 (83.33%)	3 (15.00%) 17 (85.00%)	Severity of disease Mild Moderate-severe
0.282	3 (16.67%) 15 (83.33%)	6 (30.00%) 14 (70.00%)	Duration of symptoms Intermittent Persistent
0.479^**^	48.90 ± 16.26	52.72 ± 16.68	Weight (kg) ((Mean±SD)
0.809^**^	19.72 ± 11.64	18.90 ± 14.90	Age (years) (Mean±SD)

* Based on chi-square or Fisher’s exact test ** Based on independent t-test. SD: Standard deviation

The mean±SD of TNSS in the treatment group was 8.55 ± 2.46 at the baseline and 1.16 ± 0.90 after 14 days. In the control group, it was 8.72 ± 1.99 at the baseline and 2.89 ± 1.49 after 14 days. At the baseline, there was not any significant difference regarding TNSS between the two groups (p=0.806), but after 14 days, the difference reached statistical significance (p=0.0001). In addition, the analysis revealed the group-time interaction (the change of TNSS over two weeks between the two groups) (p=0. 012), the main effect of time (within-subjects factor) (p=0.0001), and the main effect of group (between-subject factor) (p=0. 026) were statistically significant for TNSS. Also, a statistically significant decrease in TNSS was observed 14 days after initiating the interventions in both the treatment (p=0.0001) and control (p=0.0001) groups.

The prevalence of allergic rhinitis symptoms in the treatment group decreased statistically significantly 14 days after initiating the treatment in comparison with that in the control group (Table 2).

In addition, the severity of the four main symptoms of allergic rhinitis based on TNSS in the treatment and control groups at the baseline and after 14 days is presented in Table 3. 

The percentage of the patients with symptom improvement in each group is shown in Table 4. The improvement of nasal itching (p=0.042), runny nose (p=0.048) and sneezing (p=0.039) symptoms were statistically significantly different between the two groups after two weeks. Furthermore, the patients reported no adverse effects during this trial.

**Table 2:** Prevalence of allergic rhinitis symptoms during two weeks in treatment and control groups

**p-value** ^***^	**95% CI** ^*^^*^ ** for Exp (B)**	**Exp (B)** ^ *^	**Presence of symptoms(%)**	**Group**	**Time**
0.710	0.643-1.351	0.932	41.4 %	Treatment	Baseline
		1	60.5 %	Control	
0.047	0.512-0.995	0.713	12.5 %	Treatment	After 14 days
		1	27.2 %	Control	

* Exponential beta **Confidence interval ***Based on generalized estimating equation (longitudinal model)

**Table 3:** Severity of the main symptoms of allergic rhinitis based on total nasal symptom score in the treatment and control groups at the baseline and after 14 days

**Severity of symptom **	**Baseline** **Number (percent)**		**After 14 days** **Number (percent)**		**p-value** ^*^ **(Baseline)**	**p-value** ^*^ **(after 14 days)**
	**Treatment** **(n=20)**	**Control ** **(n=18)**	**Treatment** **(n=20)**	**Control** ** (n=18)**		
Nasal itching None Mild Moderate Severe	3 (15.00%) 2 (10.00%) 8 (40.00%) 7 (35.00%)	0 (0.00%) 6 (33.33%) 3 (16.67%) 9 (50.00%)	17 (85.00%) 3 (15.00%) 0 (0.00%) 0 (0.00%)	6 (33.33%) 9 (50.00%) 3 (16.67%) 0 (0.00%)	0.059	0.004
Nasal congestion None Mild Moderate Severe	1(5.00%) 2 (10.00%) 7 (35.00%) 10 (50.00%)	0 (0.00%) 2 (11.11%) 7 (38.89%) 9 (50.00%)	17 (85.00%) 2 (10.00%) 1 (5.00%) 0 (0.00%)	10 (55.55%) 5 (27.78%) 3 (16.67%) 0 (0.00%)	0.813	0.135
Runny nose None Mild Moderate Severe	1 (5.00%) 2 (10.00%) 7 (35.00%) 10 (50.00%)	0 (0.00%) 2 (11.11%) 7 (38.89%) 9 (50.00%)	19 (95.00%) 1 (5.00%) 0 (0.00%) 0 (0.00%)	11 (61.11%) 6 (33.33%) 1 (5.56%) 0 (0.00%)	0.813	0.037
Sneezing None Mild Moderate Severe	1 (5.00%) 3 (15.00%) 7 (35.00%) 9 (45.00%)	0 (0.00%) 1 (5.56%) 6 (33.33%) 11 (61.11%)	14 (70.00%) 6 (30.00%) 0 (0.00%) 0(0.00%)	5 (27.78%) 8 (44.44%) 5 (27.78%) 0 (0.00%)	0.536	0.009

* Based on chi-square

**Table 4:** The percentage of patients with symptom improvement in each group after 14 days compared with baseline

**Group**	**Runny nose **	**Nasal congestion**	**Nasal itching**	**Sneezing**	**Cough**	**All symptoms**
Control	51.1%	63 %	54 %	56 %	48 %	54.4 %
Treatment	78 %	69 %	71 %	79 %	51 %	71 %
p-value	0.048	0.211	0.042	0.039	0.631	0.047

Change was calculated by subtracting the day 14 data from the baseline data.* Based on chi-square

## Discussion

The current study findings revealed that oat had significant effects as an adjunct treatment for improving allergic rhinitis symptoms. After two weeks of intervention, the patients’ total nasal symptom scores significantly decreased in the treatment group compared with the control group. Also, the prevalence of allergic rhinitis symptoms was significantly lower in the treatment group after two weeks. The percentage of the patients who had improvement in the symptoms was higher in the treatment group (71%) in comparison with those (54.4%) in the control group after two weeks. The improvement in nasal itching, runny nose and sneezing was significant in the treatment group. Moreover, the severity of nasal itching, runny nose and sneezing symptoms was significantly declined in the treatment group compared with the control patients after two weeks. It should be mentioned that the standard treatment (oral antihistamines and glucocorticoid nasal spray) was effective in both groups (based on the paired t-test within each group), but supplementation with oat grain extract capsules was associated with significantly higher improvements in the outcomes in the treatment group. This finding demonstrated that the oat capsules increased the effectiveness of the standard treatment. These results could be related to the antihistamine, anti-inflammatory, and antioxidant effects of oat (Sang and Chu, 2017). Oat has been shown to inhibit the release of arachidonic acid from phospholipids and consequently its metabolism into prostaglandins and leukotrienes (Dawid-Pać 2013). Avenanthramides, major bioactive compounds of oat, have a structure similar to antihistamine compound tranilast and can be used to treat allergic reactions (Boz 2015). Beta-glucan, another major compound of oat, can modulate immune system function and may alleviate the symptoms of allergic diseases (Jesenak et al. 2014a). 

The use of biologically active polysaccharides like beta-glucan of natural origin is a new therapeutic strategy for the treatment and prevention of allergic diseases (Jesenak et al. 2014a). Some clinical studies have evaluated the effects of beta-glucan on relieving the symptom of allergic diseases. For instance, in a study undertaken on children aged 2 to 5 years with recurrent respiratory infections, the use of beta-glucan was associated with a significant reduction in peripheral blood eosinophilia as well as stabilization of total serum IgE levels which are both the markers of allergic inflammation. This reduction has been more pronounced in atopic individuals (Jesenak et al. 2014b). Also, in a study conducted by Talbot et al., it was found that daily supplementation with oral beta-glucan could improve the symptoms of allergy, overall physical health, emotional well-being and quality of life in individuals with self-described ragweed allergy, but it had no effect on IgE concentration (Talbott et al. 2013). In addition, Yamada et al. demonstrated that the oral administration of beta-glucan alleviated ongoing symptoms of allergic rhinitis and rhinoconjunctivitis in Japanese individuals with seasonal allergy to cedar pollen and perennial allergy (Yamada et al. 2007). In line with the above, Kirmaz et al., based on a clinical trial of 24 patients with allergic rhinitis, suggested that oral beta-glucan may be used as an adjunct to standard treatment in patients with allergic rhinitis because of its immunomodulatory effects (Kirmaz et al. 2005). Also, Miraglia Del Giudice et al. showed that nasal spray formulation of resveratrol plus carboxymethyl-β-glucan significantly reduced nasal symptoms including itching, sneezing, rhinorrhea, and obstruction as well as the use of rescue medication such as cetirizine in children with pollen-induced allergic rhinitis (Miraglia Del Giudice et al. 2014). Furthermore, it was reported that children aged 8–13 years who had high intake of whole grain products in their diet might have a protective effect against asthma. Oat is also a whole grain and may elicit similar beneficial effects (Sang and Chu 2017; Tabak et al. 2006). 

Numerous studies have also demonstrated clinical benefits of topical oat formulations in the treatment of a variety of inflammatory dermatologic disorders like dry skin, atopic dermatitis, eczema, and pruritic skin diseases (Mengeaud et al. 2015; Reynertson et al. 2015; Nakhaee et al. 2015; Shohrati et al. 2017; Sobhan et al. 2020). Reynertson et al. conducted a clinical study on 29 subjects with bilateral mild to moderate itching and moderate to severe dry skin on their lower legs to investigate the anti-inflammatory activities of oatmeal in the treatment of pruritus associated with dry and irritated skin. It was reported that after using oat lotion, dryness, redness, cracks, and skin rashes improved in all the patients. In this study, it was shown that the anti-inflammatory and antioxidant effects of oat extract were related to reduced nuclear factor kappa B (NF-κB) activity, reactive oxygen species (ROS) and interleukin-8 (IL-8) production (Reynertson et al. 2015). Along the same lines, Mengeaud et al. reported that 3 months of maintenance therapy with a sterile, preservative-free emollient cream containing oat led to a significant improvement in atopic dermatitis clinical symptoms in children (Mengeaud et al. 2015). In addition, Shohrati et al. demonstrated that topical extract of *Avena sativa* L. in sulfur mustard-exposed patients with chronic pruritus decreased pruritus, but topical betamethasone was more effective in all aspects 4 weeks after the start of the study (Shohrati et al. 2017). Similarly, Sobhan et al. showed that colloidal oatmeal cream as an adjunctive treatment in combination with topical corticosteroids in the patients with chronic irritant hand eczema could improve symptoms and pruritus (Sobhan et al. 2020). Furthermore, Nakhaee et al. reported that *Avena sativa* L. lotion could decrease uremic pruritus (Nakhaee et al. 2015). 

In the current study, no side effects were reported by the patients due to oral intake of the oat capsules. Other studies using oral oat also did not observe any side effects. It should be mentioned that flatulence and anal irritation were noted as oat side effects. So, it was recommended that oat products should be taken with plenty amounts of water to disperse the fiber well in the bowel and to reduce gastrointestinal side effects (Al-Snafi 2015).

The limitations of this clinical trial were relatively small sample size and short duration. Also, it is suggested to conduct similar studies with an increased sample size, longer duration, more frequent follow-ups, different doses, different dosage forms like nasal formulations, use of oat capsules as an initial treatment not as an adjunct therapy, performance of laboratory tests to evaluate the response to treatment along with evaluating clinical symptoms, and performing pharmacokinetic studies in patients during the intervention period.

It was concluded that the hydroalcoholic extract of oat grains (*Avena sativa* L.) capsules was an effective adjunctive therapy to improve allergic rhinitis symptoms. Yet more researches are required to confirm these findings.

## Abbreviations

IgE: Immunoglobulin E

°C: Degree Celsius

SD: Standard Ceviation

λmax: Wavelength of Maximum Absorbance 

RSD: Relative Standard Deviation

mg: Milligram

ml: Mililiter

USP: U.S Pharmacopeia

TNSS: Total Nasal Symptom Score

NF-κB: Nuclear Factor Kappa B

ROS: Reactive Oxygen Species

IL-8: Interleukin-8
